# Application of functional regression in biomechanical assessment of neck disability: Selection of clinically relevant variables

**DOI:** 10.1371/journal.pone.0340428

**Published:** 2026-02-19

**Authors:** Elisa Aragón-Basanta, Guillermo Ayala, Pilar Serra-Añó, Álvaro Page

**Affiliations:** 1 Instituto Universitario de Ingeniería Mecánica y Biomecánica, Universitat Politècnica de València, Valencia, Spain; 2 Departament of Statistics and Operation Research, Universitat de València, Burjasot, Spain; 3 Departament of Physiotherapy, Universitat de València, Valencia, Spain; University of Perugia: Universita degli Studi di Perugia, ITALY

## Abstract

**Background**: The Neck Disability Index (NDI) is widely used to assess neck pain-related disability. It is commonly combined with objective measures, particularly range of motion and peak velocity, but these variables usually show weak correlations with patient-reported outcomes. Functional data analysis (FDA) makes it possible to analyze complete kinematic waveforms and provides tools to identify relevant motion features most related to disability.

**Objective**: This study aimed to examine whether scalar-on-function regression of cervical angular velocity curves can predict NDI scores in patients with nonspecific neck pain and which motion variables and sub-phases contribute most to the prediction.

**Methods**: We analyzed 56 recordings from 28 patients, each paired with an NDI questionnaire, which were collected over two sessions. Cervical flexion–extension, lateral flexion, and axial rotation velocities were processed using functional principal component analysis to reduce dimensionality while retaining the main modes of variation.

Univariate and bivariate scalar-on-function regression models were estimated. Model selection was based on a procedure that combined the Akaike Information Criterion, Bayesian Information Criterion, and measures of goodness-of-fit, ensuring a balance between model simplicity and predictive accuracy. Comparisons were made using non-functional regressions, including demographic and anthropometric variables.

**Results**: The bivariate flexion–extension plus lateral flexion model showed the best performance (r = 0.677), which was clearly higher than that of the non-functional regressions (r ≤ 0.391). The coefficient functions indicated that the acceleration and deceleration phases were the most informative in explaining variability in NDI scores. The inclusion of anthropometric variables did not improve model performance.

**Conclusions**: Functional regression of velocity curves improves the prediction of NDI scores and highlights specific phases of movement that are clinically relevant. Larger studies are required to confirm these findings and assess their clinical applicability.

## Introduction

Functional data analysis (FDA) represents a substantial advancement in the clinical application of biomechanics, facilitating the correlation of kinematic and dynamic variables with health conditions and functional changes. Unlike traditional numerical variables, which rely on extreme values or phase durations, functional variables use entire curves to characterize the movement. FDA offers significant advantages over classical statistical methods by preserving all information associated with continuous movement, rather than reducing it to a few discrete values. Furthermore, functional variables do not require the identification of specific points or characteristics of the curve, such as maxima, minima, or specific times, which can be challenging to discern when dealing with abnormal movement patterns associated with injury or disability [1–3].

There is substantial scientific literature on applying FDA in biomechanics [3–5], often focusing on comparing healthy and diseased subjects [6,7]. These studies provide valuable insights into how movement patterns change in individuals with disabilities. However, the differences between healthy and affected individuals do not always directly correlate with continuous changes in injury severity or pain, both of which are crucial for analyzing disease progression and the effectiveness of rehabilitation treatments [8,9]. Alternatively, in patient cohorts, regression models offer a more appropriate strategy by uncovering relationships between biomechanical variables and disability measured via clinical or pain scales [10]. Most current studies rely on correlations between numerical variables and clinical scale scores, which typically demonstrate weaker associations than those derived from healthy–ill comparisons [11–13]. By contrast, functional regression models demonstrated stronger predictive performance. In particular, scalar-on-function regression models allow the identification of relevant functional variables that can predict a patient’s functional state [14]. Although this type of model has received considerable theoretical attention [3,15], its translation into routine clinical assessment remains limited [14,16].

A limitation of scalar-on-function regression models is their inherent high dimensionality, which can lead to overfitting [3,15]. Even with dimensionality reduction via basis expansion, analyses must include numerous kinematic or dynamic variables (e.g., position, velocity, force) across multiple joints or movements, resulting in dozens of potential predictors. Selecting the most relevant variables thus demands methods capable of simultaneously evaluating multiple predictors. It is therefore essential to select an appropriate basis that preserves relevant information while minimizing dimensionality. Traditional criteria based on multiple correlations are insufficient and vulnerable to overfitting. Instead, penalized model selection criteria such as the Akaike Information Criterion (AIC) or Bayesian Information Criterion (BIC)—which balance goodness of fit with model complexity—are well suited to choose both the basis and relevant predictors by achieving a compromise between the simplicity of the model and the goodness of fit [17,18]. Unfortunately, despite their suitability, these criteria are still rarely used in clinical applications of functional regression [14,19].

In this study, we propose and validate a novel approach using functional regression models to identify relevant kinematic variables in the functional assessment of nonspecific neck pain. Our work addresses a significant gap in the literature by directly correlating complete movement curves with clinical disability indices. Neck injuries are second only to lumbar injuries in prevalence, making kinematic analyses of the cervical spine valuable indicators of neck joint function [20–22]. However, most published studies do not use a functional approach, which has the potential to provide deeper insight into the relationships between kinematic variables and levels of disability or pain. Typically, these studies relate scalar summaries such as ranges of motion or angular velocities in the three anatomical planes to pain scales [23] or to disability scales [24], among others [8,12,13,20–22].

This study uses a scalar-on-function regression model with the Neck Disability Index (NDI) as the response variable. Predictor variables include angular position and angular velocity curves from flexion-extension (FE), lateral flexion (LF), and axial rotation (AR), as well as control covariates like sex, age, neck length, head mass, and neck mass. Six functional variables and five numerical variables are used, which require combining quality criteria, in addition to the classic ones based on the correlation coefficient and p-values to select the most predictive and robust models. The quality of the models and the risk of overfitting were analyzed to provide criteria for selecting the base dimension. Finally, we compared multiple functional regression models to identify which kinematic variables and movements are most strongly associated with the NDI.

## Materials and methods

### Materials

The present analysis used kinematic data from the participants of a previously published randomized controlled trial on cervical spine manipulation in patients with nonspecific neck pain [25] (ID: NCT04059692). In the original trial, conventional numerical variables such as range of motion (RoM) and peak velocity were analyzed. However, the continuous movement recordings obtained during the same sessions were also preserved, and in this study we processed and analyzed those waveforms using Functional data analysis (FDA). Twenty-eight participants contributed to two sessions (pre- and post-treatment), resulting in 56 observations. For the present analysis, both sessions were pooled to provide two data points per subject. The current study focused exclusively on the association between NDI and kinematic variables, and no pre–post comparisons or treatment effects were considered.

#### Participants and eligibility criteria.

The inclusion criteria were as follows: age between 18 and 65 years, acute or chronic nonspecific neck pain lasting at least one month, pain intensity ≥ 3 on a visual analog scale and NDI ≥ 5 in the first session. Exclusion criteria included inflammatory or rheumatic disease; vestibular disorders; neurological conditions; recent trunk or shoulder surgery; or the use of opioids, antidepressants, or sedatives. All participants provided written informed consent and the study protocol was approved by the Ethics Committee of the University of Valencia (H1450106985729).

#### Kinematic assessment.

**Fig 1:**
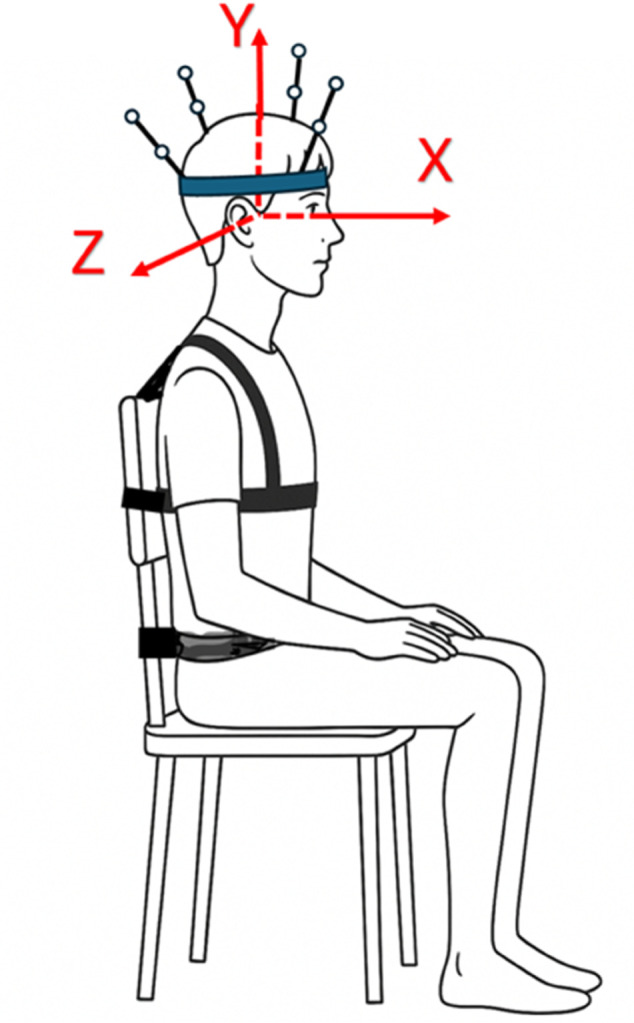
Experimental setup. Participants sat with the trunk secured to the backrest to limit torso motion. Head motion was recorded using eight markers attached to a rigid helmet. The local reference frame was centered at the midpoint between both tragus points. The X-axis (lateral flexion) pointed anteriorly through the line connecting the infraorbital landmarks, the Z-axis (flexion extension) connected the tragus points toward the right side, and the Y-axis (axial rotation) completed the right-handed triad. Additional details on the determination of anatomical axes are described in Venegas et al. [27].

In each session, the participants performed cyclic cervical movements of flexion–extension (FE), right and left lateral flexion (LF), and right axial rotation (AR) in randomized order. Movements were recorded using the Kinescan-IBV videophotogrammetry system [26] while the participants sat in a custom-designed chair with trunk stabilization (Fig 1). To minimize torso movements during the trials, participants were instructed to avoid compensatory trunk actions. In addition, the torso was stabilized using two straps attached to the chair, securing the shoulders and ensuring that the recorded motion originated solely from the neck. A rigid headband with reflective markers was used to record head movements.

Calibration was performed with additional markers placed on the right and left tragus, the nasal bone, and the infraorbital points to define the anatomical reference system; these calibration markers were removed immediately afterward. The experimental procedure and the determination of the anatomical axes followed the methodology described in Venegas et al. [27].

Each participant completed seven continuous cycles per movement during each session (before and after treatment), with the first and last cycles discarded. Time scales were normalized, and the functional mean of the five remaining position–velocity curves was calculated. Additional numerical variables were derived, such as range of motion (RoM) and velocity ranges for movement description [26].

#### Anthropometric measurements.

Neck length was measured as the straight-line distance between the marker placed on the spinous process of C7 and the midpoint between the tragus markers, which defined the origin of the anatomical reference system in the neutral head position. Head and neck masses were estimated using regression equations proposed by [28], specifically designed to provide anthropometry-based estimates of the inertial parameters of the head–neck complex.

#### Disability assessment.

The Spanish version of the Neck Disability Index (NDI) was used to assess disability and served as the dependent variable for each observation [29]. The questionnaire, with a score of 0-50, was administered prior to both measurement sessions.

### Methods

#### Functional linear regression model.

Our data consist of $\left(x_{i},y_{i}\right)$ where *x*_*i*_ contains functional and scalar predictors and *y*_*i*_ the corresponding response. The scalar-on-function regression models the conditional distribution of *Y*_*i*_ given the (scalar and functional) predictors *x*_*i*_. For illustration, consider the simplest case with one functional predictor *x*_*i*_. The model is

$$Y_{i}=\beta_{0}+\int \beta_{1}(t)x_{i}(t)dt+\epsilon_{i},$$ (1)

with random errors $\epsilon_{i}$’s are independent and identically distributed with $\epsilon_{i}\sim N(0,\sigma^{2})$. We assume that $\int_{T}x^{2}(t)dt<\infty$ i.e. the square of the function is integrable.

To facilitate estimation, expand $\beta_{1}$ and *x*_*i*_ in appropriate bases: $\beta_{1}(t)=\sum_{k_{1}=1}^{K_{\beta_{1}}}b_{k_{1}}\theta_{k_{1}}(t)$ and $x_{i}(t)=\sum_{k_{2}=1}^{K_{x}}c_{ik_{2}}\psi_{k_{2}}(t)$ in such a way that

$$\int_{T}\beta_{1}(t)x_{i}(t)dt=\int_{T}\sum_{k_{1}=1}^{K_{\beta_{1}}}b_{k_{1}}\theta_{k_{1}}(t)\sum_{k_{2}=1}^{K_{x}}c_{ik_{2}}\psi_{k_{2}}(t)dt\;=\sum_{k_{1}=1}^{K_{\beta_{1}}}\sum_{k_{2}=1}^{K_{x}}b_{k_{1}}c_{ik_{2}}\int_{T}\theta_{k_{1}}(t)\psi_{k_{2}}(t)dt=\sum_{k_{1}=1}^{K_{\beta_{1}}}\sum_{k_{2}=1}^{K_{x}}c_{ik_{2}}J_{k_{1}k_{2}}b_{k_{1}}=\sum_{k_{1}=1}^{K_{\beta_{1}}}v_{k_{1}}b_{k_{1}},$$ (2)

where $v_{k_{1}}=\sum_{k_{2}=1}^{K_{x}}c_{ik_{2}}J_{k_{1}k_{2}}$ is known. The multiple regression model would be

$$Y_{i}=\beta_{0}+\sum_{j=1}^{K_{\beta_{1}}}v_{j}b_{j}+\epsilon_{i},$$ (3)

where the *b*_*j*_’s are the coefficients. The coefficients *c*_*ik*_ are estimated using the least squares method. The problem is reduced to a multiple regression model.

If several functional predictors $x_{i}^{\left(s\right)}$ with s=1,…,S are considered besides scalar predictors $u=(u_{1},\ldots ,u_{q})\in R^{q}$ i.e. $x_{i}=(x_{i}^{\left(1\right)},\ldots ,x_{i}^{\left(S\right)},u_{1},\ldots ,u_{q})$ then the functional regression model can be formulated as

$$Y_{i}=\beta_{0}+\sum_{s=1}^{S}\int x_{i}^{\left(s\right)}(t)\beta_{s}(t)dt+\sum_{r=1}^{q}\beta_{r}^{\left(u\right)}u_{ir}+\epsilon_{i}.$$ (4)

It can be chosen different basis functions for the different coefficient functions $\beta_{s}$. Additionally, note that the functional predictors have to be expressed in its own basis.

To compare modeling approaches, we fitted standard regression models using scalar predictors. For each movement, the model included the range of motion (RoM), the range of angular velocity (RoV), and their interaction term (RoM × RoV). These models serve as benchmarks against which we compare our functional regression results. Including both RoM and RoV, along with their interaction, is justified by network analysis findings that demonstrate strong statistical interactions between these variables [8].

#### Dimensionality reduction via functional principal component analysis.

We represented the functional data —each movement curve— using a B-spline basis of 40 functions. To reduce dimensionality, we subsequently applied Functional Principal Component Analysis (FPCA), which generalizes standard PCA to functional data by identifying the dominant modes of variation across the movement curves. FPCA allows any curve to be approximated as the mean function plus a linear combination of these functional principal components [30–34].

Since some analyses involve more than one functional variable, we also employed bivariate FPCA (bFPCA). This technique performs joint FPCA on a pair of curves, capturing both shared and complementary modes of variation across them. Bivariate and multivariate FPCA are increasingly used in biomechanics when dealing with simultaneous functional signals, for example, in sports biomechanics to analyze multivariate movement signatures [35,36]. More information about FPCA or bFPCA can be found in [1].

To select the optimal number of functional principal components, we evaluate multiple model quality metrics:

- Akaike Information Criterion (AIC) [17], computed as $-2\log\hat{L}+2k$, where $\hat{L}$ is the maximum likelihood of the model, and *k* is the number of parameters.
- Bayesian Information Criterion (BIC) [18], computed as $-2\log\hat{L}$  +  klog(n), penalizing complexity more heavily for larger sample sizes (n).
- The multiple linear correlation coefficient (*r*) and the F-statistic with associated p-value to assess model significance.

In addition to AIC, BIC, the correlation coefficient, the F statistic, and the p-value, the cumulative explained variance was also considered, to ensure that a substantial proportion of the information contained in the original curves was preserved. Specifically, we ensured that the number of components retained accounts for at least 95% of the total variance.

Our strategy for selecting the number of components was as follows:

1. Increase the number of components until cumulative explained variance reaches 95%.
2. Assess each model’s predictive accuracy using high *r* and significant F-test.
3. Among models meeting these criteria, choose the one that minimizes AIC and BIC, striking a balance between goodness of fit and model parsimony.

Seventeen models—ranging from 4 to 20 components—were fitted per functional predictor to identify optimal dimensionality. These criteria also allowed comparison across movement types (flexion-extension, lateral flexion, axial rotation) and data types (angle vs angular velocity). For cases with both angle and velocity curves, bFPCA was used, and component selection followed the same scheme to determine the most predictive movement patterns.

#### Model comparison and variable selection.

Once the FPCA-based functional models were selected (with optimal number of components per criteria such as cumulative explained variance, AIC, BIC, *r*, F-statistic and p-value), we proceeded in two steps.

1. Comparison of functional models. We compared functional regression models using either angle curves or angular velocity curves for each movement, including flexion–extension, lateral flexion, and axial rotation. For angle–velocity pairs, we also applied bivariate FPCA. Each model’s performance was evaluated using the standard criteria (AIC, BIC, *r*, F-statistic, p-value) to determine which combination of movement and kinematic variable yields the highest predictive power.
2. Demographic and anthropometric covariates. Starting from the best functional model for each movement domain, we tested whether adding scalar covariates (sex, age, neck length, head mass, and neck mass) improved the model performance. For each scalar predictor, a separate extended model was fitted and compared again using AIC, BIC, the correlation coefficient, the F-statistic, and the p-value for the null hypothesis that the added predictor had no effect. A low p-value (e.g. <0.05) indicates significant improvement over the base functional model.

As a baseline for comparison, we also fitted simple scalar-only models for each movement using the range of motion (RoM), range of angular velocity (RoV), and their interaction (RoM × RoV) as the predictors. This enables a direct assessment of whether our functional regression models offer superior predictive performance compared to traditional models built on these scalar movement summaries (RoM and RoV) [14].

All analyses were conducted in R using the fda and fda.usc packages.

## Results

### Participants and kinematic data

The sample comprised 28 subjects (18 women and 10 men) with non-specific neck pain diagnosed by experienced physiotherapists, measured for flexion-extension, lateral flexion, and axial rotation movements, each assessed twice, before and after neck treatment. Therefore, there were 56 samples. However, only 55 were considered because one has been discarded owing to errors during the measurement process.

Some basic numerical characteristics of the sample are displayed in Table 1, where the mean and standard deviation of some variables of the subjects by sex are shown. The NDI scores ranged from 1 to 19.

**Table 1:** Mean and standard deviation of the variables NDI, age (years), weight (kg), height (cm), neck length (cm), head mass (kg), and neck mass (kg) by gender.

Variable	Women (N=35)	Men (N=20)	Total (N=55)
	Mean (sd)	Mean (sd)	Mean (sd)
NDI	11.11 (4.94)	8.35 (3.96)	10.11 (4.76)
Age (years)	35.46 (12.21)	33.30 (13.06)	34.67 (12.45)
Weight (kg)	65.06 (9.99)	83.58 (11.59)	71.79 (13.82)
Height (cm)	162.69 (6.80)	178.8 (7.15)	168.55 (10.41)
Neck length (cm)	14.50 (1.77)	16.15 (0.97)	15.10 (1.72)
Head mass (kg)	3.71 (0.41)	4.44 (0.49)	3.98 (0.56)
Neck mass (kg)	1.40 (0.17)	1.78 (0.15)	1.54 (0.24)

**Fig 2:**
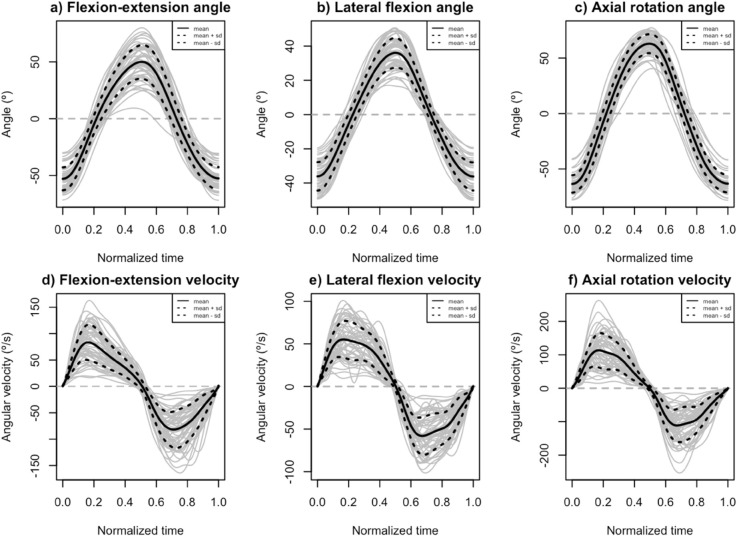
Kinematic curves. Angular position of (a) flexion-extension; (b) lateral flexion; (c) axial rotation. Angular velocity of: (d) flexion-extension; (e) lateral flexion; (f) axial rotation. The grey lines are the curves for each subject, with the functional mean (black) plus and minus one functional standard deviation (dotted black).

In addition, Fig 2 shows the curves for the 55 repetitions (gray) for angle (top) and angular velocity (bottom) in the flexion-extension, lateral flexion, and axial rotation movements, together with the functional mean (black) plus and minus one standard deviation (dotted black).

Table 2 shows the values of range of motion (RoM) and angular velocity (RoV) for each of the three movements analyzed: flexion-extension, lateral flexion and axial rotation.

**Table 2:** Mean and standard deviation of the range of angular motion (RoM) and range of angular velocity (RoV) for flexion-extension, lateral flexion and axial rotation.

Movement	RoM (^°^)	RoV (^°^/s)
	Mean (sd)	Mean (sd)
Flexion-extension	103.75 (20.84)	173.38 (67.95)
Lateral flexion	72.61 (16.20)	122.50 (40.35)
Axial rotation	127.10 (14.03)	247.03 (95.67)

### Non-functional regression models

Three classical (non-functional) linear regression models were implemented for each movement, whose predictors were range of motion (RoM), range of angular velocity (RoV) and their interactions. Table 3 shows values of the multiple linear correlation *r*, F statistic and p-value. The highest value of the multiple correlation coefficient was for the lateral flexion movement (0.391), followed by flexion-extension (0.356), and axial rotation, with the lowest value (0.296). On the other hand, only the models for the lateral flexion and axial rotation movements had a significant F statistic (0.036 in both cases), while for flexion-extension, the p-value was 0.073.

**Table 3:** Multiple linear correlation coefficient value *r*, F-statistic, and p-value of the scalar models for the three movements: flexion-extension, lateral flexion, and axial rotation. In all models, the predictors used were range of motion (RoM), range of velocity (RoV), and their interactions.

Movement	*r*	F	p-value
Flexion-extension	0.356	2.460	0.073
Lateral flexion	0.391	3.072	0.036
Axial rotation	0.296	1.634	0.036

### Functional regression models

#### Optimal number of functional principal components.

**Fig 3:**
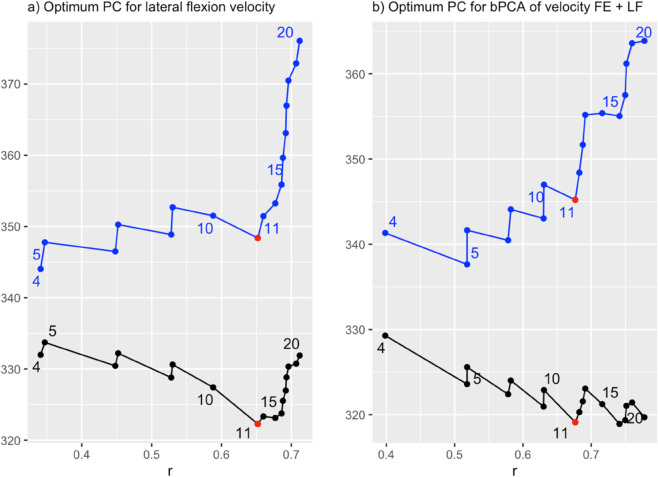
Selection of the optimal number of principal components. The plot shows the multiple linear correlation coefficient (x-axis) and the AIC and BIC (y-axis) according to different numbers of principal components, varying from four (first point) to 20 (last point). Black and blue represent the AIC and BIC values, respectively. The red point corresponds to the optimal number of principal components chosen (11 for both models). (a) The plot on the left corresponds to the functional regression model, whose predictor is the lateral flexion velocity. (b) The one on the right is for the principal components model concatenating the flexion-extension and lateral flexion velocity curves.

To illustrate the process of selecting the optimal number of basis functions, Fig 3 displays the evolution of key model quality metrics for two representative functional regression models. The first model includes a single functional predictor—lateral flexion velocity—while the second uses two functional predictors—velocities of flexion-extension and lateral flexion—. In both cases, the figure plots AIC (black) and BIC (blue) against the multiple linear correlation coefficient *r*, shown on the x-axis. Each point on the plot corresponds to a model fitted with a specific number of principal components, ranging from 4 (first point) to 20 (last point), so that the plot captures how AIC, BIC, and *r* vary as component count increases.

In the left panel, applying the elbow method reveals that both AIC and BIC reach their minimum at 11 principal components, which is highlighted in red as the optimal choice.

However, not all models displayed such a clear minimum. The right panel of Fig 3 provides an illustrative example: the model using both functional predictors shows that while AIC also attains a minimum at 15 components and *r* continues to improve, the BIC rises sharply. In such a case, choosing 11 components remains preferable due to the more favorable trade-off between fit and parsimony.

#### Model comparison.

Table 4 shows the values of explained variability, AIC, BIC, multiple correlation coefficient, F statistic, and p-value for each model with the optimum number of principal components chosen for each movement. In all cases, the explained variability was approximately 99%. Among the three movements studied, the optimal number of principal components for angle-based models and for flexion–extension (FE) velocity was nine. In contrast, for both lateral flexion (LF) and axial rotation (AR) velocity, the optimal number was eleven principal components.

**Table 4:** Model comparison. Optimal number of functional principal components, proportion of variance explained, Akaike Information Criterion (AIC), Bayesian Information Criterion (BIC), multiple correlation coefficient (*r*), and F statistic with associated p-value for each model.

Model	Optimum number PC	Variability explained %	AIC	BIC	*r*	F	p-value
Angle FE	9	99.9	330	352	0.535	2.002	0.061
Angle LF	9	99.9	329	351	0.555	2.222	0.038
Angle AR	9	99.8	344	367	0.275	0.409	0.924
**Velocity FE**	9	99.7	324	346	0.603	2.851	0.010
**Velocity LF**	11	99.7	322	348	0.652	2.897	0.006
Velocity AR	11	99.6	340	366	0.463	1.065	0.410
**Velocity FE+LF**	11	98.7	319	345	0.677	3.300	0.002

The predictive performance of the models based on angular displacement was limited. Specifically, the multiple linear correlation coefficients (*r*) were relatively low: 0.535 for flexion–extension (FE), 0.555 for lateral flexion (LF), and 0.275 for axial rotation (AR). However, these values were higher than those observed for the traditional scalar models based on the range of motion (RoM) and range of velocity (RoV), which yielded *r* values of 0.356 for FE, 0.391 for LF, and 0.296 for AR (Table 3). Additionally, these angular models produced the highest AIC and BIC values among all functional models. Only the LF model reached statistical significance (*p* = 0.038).

In contrast, models using angular velocity as a functional predictor demonstrated markedly improved performance. The FE and LF velocity models achieved substantially higher correlation coefficients (*r* = 0.603 and *r* = 0.652, respectively) and both were statistically significant (*p* = 0.010 and *p* = 0.006). These models also recorded the lowest AIC and BIC values among all single-variable functional models. The AR velocity model, on the other hand, performed worse; it yielded *r* = 0.463, with a not significant p-value (*p* = 0.410), and displayed higher AIC and BIC values.

Building on these findings, a combined model was formulated by concatenating the velocity curves of FE and LF and applying bivariate functional principal component analysis (bFPCA). As reported in the last row of Table 4, this bFPCA model—with 11 optimal components—delivered the most favorable metrics of all models evaluated: AIC = 319, BIC = 345, *r* = 0.677, and a highly significant *p*-value of 0.002. This model was selected as the basis for the subsequent analysis involving demographic and anthropometric covariates.

#### Effect of scalar covariates.

To evaluate the impact of demographic and anthropometric covariates, we began with the best-performing functional model as the baseline: the bFPCA model with 11 components combining flexion–extension and lateral flexion velocities. We then added each scalar predictor—sex, age, neck length, head mass, and neck mass—individually to assess whether any would enhance the model’s predictive ability.

Table 5 reports the AIC, BIC, multiple linear correlation coefficient *r*, F-statistic, and p-value for testing the null hypothesis that the coefficient of the scalar predictor is zero. The first row corresponds to the base functional model, while subsequent rows present the results for each model, extended by a single covariate. In all extended models, both AIC and BIC increased relative to the baseline, indicating that model parsimony did not improve. The multiple correlation coefficient *r* increased slightly in all cases; the most notable increase occurred with the inclusion of sex, raising *r* from 0.677 to 0.693 (increase of 0.016). However, none of the added covariates achieved statistical significance, as evidenced by non-significant p-values across all models. Therefore, there is no evidence to reject the null hypothesis, and the addition of any single scalar variable does not meaningfully improve the base functional model.

**Table 5:** AIC, BIC, multiple linear correlation coefficient *r*, F statistic, and p-value of the linear regression models. Different scalar predictors were added to the base model (only functional) to analyze whether their introduction improved the initial functional model.

Model	AIC	BIC	*r*	F	p-value
bPCA (FE+LF)	319	345	0.677	-	-
bPCA (FE+LF) + sex	319	347	0.693	1.840	0.182
bPCA (FE+LF) + age	321	349	0.679	0.223	0.639
bPCA (FE+LF) + neck length	321	349	0.677	0.017	0.896
bPCA (FE+LF) + head mass	320	349	0.682	0.554	0.461
bPCA (FE+LF) + neck mass	320	348	0.682	0.587	0.448

#### Interpreting the coefficient function *β*(t).

Since none of the scalar predictors significantly improved the performance of the initial functional model, our interpretative focus was set on the 11-component bFPCA model that combined flexion–extension and lateral flexion velocities as the baseline. To elucidate the interpretative value of the coefficient function β(t) we compared the flexion–extension (FE) and lateral flexion (LF) curves of two participants: one with a low Neck Disability Index (NDI = 4) and the other with a higher NDI (NDI = 15), both exhibiting minimal residual error to ensure a clean comparison.

**Fig 4:**
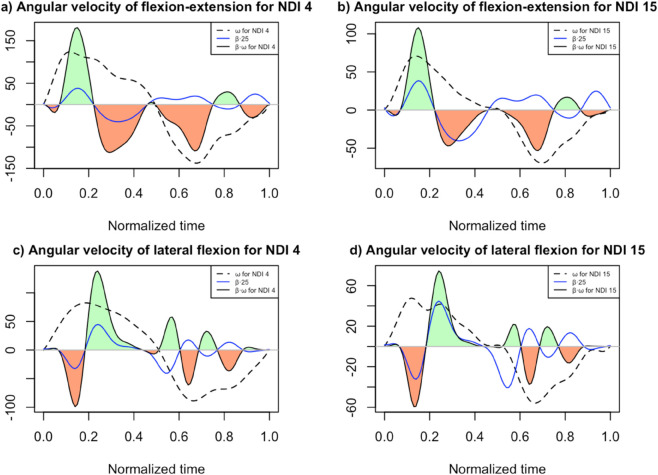
Coefficient and product functions of the functional regression model. This plot shows the coefficient and product functions of the chosen bivariate flexion-extension (top) and lateral flexion (bottom) principal component models for 11 principal components. The flexion-extension velocity curves are plotted for a participant with a low NDI of 4 (a) versus one with a high NDI of 15 (b); analogously for lateral flexion (c) and (d). In each plot, the velocity of the subject (dashed black), coefficient function (blue), and product of the velocity by the coefficient function (black) are shown. The negative and positive areas under the product function are shaded in orange and green, respectively. It is important to note that although the coefficient function is continuous from the start of the flexion extension velocity to the end of the lateral flexion velocity, as it is for the bPCA model, it has been separated for each velocity for a better interpretation of the results. The coefficient function is multiplied by 25 for better visualization in the same plot. The vertical scales have been adjusted to highlight the relative differences between patients.

This comparison is visualized in Fig 4. The top panels correspond to flexion-extension (FE) velocities, and the bottom panels correspond to lateral flexion (LF). On the left, panels (a) and (c) display data for the low-NDI subject; on the right, panels (b) and (d) show the high-NDI subject. Each panel includes the observed velocity curve (dashed black), estimated coefficient function β(t) (blue), and their pointwise product β(t)ω(t) (solid black). The coefficient function was scaled by a factor of 25 to enable a clearer joint visualization on the same plot. The vertical scales have been adjusted to highlight the relative differences between patients.

Although the coefficient function is displayed separately for FE and LF for interpretative clarity, both segments belong to a single regression model in which flexion-extension and lateral flexion velocities enter simultaneously. Thus, the model yields a unified coefficient function β(t), which is partitioned into FE- and LF-related intervals solely for visualization purposes.

According to Eq 1, the predicted NDI is obtained by adding to the model intercept $\beta_{0}$ the integral of the product function β(t)ω(t). This integral represents the area under the curve. In this way, larger positive areas (shaded in green in Fig 4) and smaller negative areas (shaded in orange) increase the predicted NDI value.

This effect is clearly observed in the upper part of Fig 4 during the flexion-extension movement, where the higher-NDI subject shows relatively smaller negative areas, leading to a net increase in predicted NDI compared with the lower-NDI subject. These differences were most evident during the braking phase of extension, immediately after reaching the peak extension velocity, and during the acceleration phase at the onset of flexion, before maximum flexion velocity is attained. In contrast, for LF, the differences between subjects were much less pronounced. The positive and negative areas of the product function are more balanced, and their relative contribution to the estimated NDI is therefore smaller than in the case of FE.

## Discussion

The evaluation of disability in patients with neck pain mainly relies on patient-reported outcomes, particularly the Neck Disability Index (NDI). Complementary information can be obtained from biomechanical measures, such as position, velocity, acceleration, or force curves, which describe the dynamics of cervical motion [10,14,20].

Most published studies compare patients with healthy controls, which is informative but does not capture the progression of pathology or the effects of rehabilitation [6,7]. Greater clinical value arises from cohort-based studies, in which biomechanical variables are directly related to indicators of functional status such as disability scales [11–13]. However, such studies remain relatively scarce, particularly those employing functional data analysis (FDA), despite its ability to exploit complete waveforms and its demonstrated potential to reveal associations with disability [14,19]. Among the FDA approaches, scalar-on-function regression models are particularly valuable, as they not only quantify the relationship between disability and complete motion curves but also identify the specific phases of movement that are most strongly associated with disability [14,16].

Functional regression models are affected by the high dimensionality of functional variables, which can lead to overfitting when an excessive number of basis functions are used for the coefficient functions. To mitigate this risk, it is necessary to implement a selection strategy that determines both the most relevant predictors and the appropriate number of basis functions, ensuring a balance between predictive accuracy and model robustness. In this study, principal component analysis was used to reduce dimensionality, and the number of components was chosen according to complementary criteria: the Akaike and Bayesian information criteria (AIC and BIC), the multiple correlation coefficient, the explained variance, and the significance of the F statistic. Increasing the number of components improves the correlation coefficient, but it also worsens the information criteria (Fig 3). Because BIC penalizes complexity more strongly than AIC, it provided a stricter reference when the two diverge. Applying these criteria to our patient cohort—characterized mainly by low to moderate levels of disability—consistently indicated an optimal solution with 9 to 11 components (Table 4).

Based on the optimal number of components selected for each model, the functional regressions consistently outperformed the nonfunctional ones. Correlation values reached up to r = 0.677 for the functional models (Table 4), whereas the non-functional regressions did not exceed r = 0.4 even when including the range of motion, range of velocity, and their interaction (Table 3). Previous work reported this advantage for flexion–extension alone [14]; the present study extends this finding by demonstrating that the superiority of functional models also applies to lateral flexion and axial rotation. The higher predictive values obtained with functional regression emphasize the methodological advantage of analyzing complete waveforms rather than summary measures.

Angular velocity showed a stronger relationship with disability than angular displacement in all three movements. Among the angle functions, only lateral flexion was significantly associated with NDI. These findings are consistent with previous studies using both numerical [8,12,13,20–22] and functional variables [14], which also reported greater predictive capacity for velocity measures.

Flexion-extension and lateral flexion were more strongly related to the NDI than axial rotation, whose position and velocity variables showed weak and non-significant associations. Between the two, lateral flexion yielded slightly higher correlations. As summarized in Table 4, the highest predictive values corresponded to models based on velocity curves of flexion-extension and lateral flexion, indicating that these movements provide the most informative predictors of disability. Although studies based on numerical variables have reported inconsistent results depending on movement, pathology, and severity, our analysis demonstrates that functional regression consistently prioritizes flexion-extension and lateral flexion.

This comparison of variables and movements highlights the strength of the proposed procedure: combining information criteria with classical fit indices makes it possible to identify the predictors that are most strongly associated with disability. In our analyses, anthropometric and demographic variables (sex, age, neck length, head mass, and neck mass) did not significantly enhance the velocity-based model. Although age is known to influence cervical mobility, its effect was likely already captured by the functional predictors, and the relatively young composition of the sample limited our ability to detect age-related changes that typically emerge in older populations [37].

A key advantage of functional regression is that the coefficient functions indicate which phases of motion are most associated with disability. This information can be visualized through the product β(t)ω(t), where the predicted NDI depends on the balance between positive and negative areas under the curve (Fig 4). Positive areas (green) increase the predicted disability, whereas negative areas (orange) reduce it. Fig 4 shows this for two representative cases: one with a low NDI (4) and one with a higher NDI (15).

For flexion-extension (upper panels), the main contributions are concentrated in three phases: a positive area around the peak velocity when the head passes through the neutral position during extension and two negative areas, one during braking before maximum extension and another during re-acceleration afterwards. In the participant with higher disability, the angular velocity was globally reduced; however, the reduction was not proportional across phases. The decrease in the positive peak was moderate, whereas the negative lobes in the braking and re-acceleration were relatively more attenuated. This disproportionate reduction shifts the balance of the integral toward a net positive value, thereby leading to a higher predicted NDI value. Clinically, this pattern suggests that increased disability is linked to lower absolute velocities during braking and acceleration around end-range extension, which reduces the protective negative contributions and increases disability scores.

For lateral flexion (lower panels), the differences between participants with relatively lower and higher disability were less pronounced. Several positive and negative areas were observed; however, their overall effect was smaller than that of flexion–extension. In the bivariate model, lateral flexion primarily modulated the predicted NDI, acting as a secondary adjustment to the dominant influence of flexion-extension curve rather than determining it independently.

Taken together, these results illustrate how functional regression not only improves predictive performance but also provides interpretable information about the phases of motion most closely associated with disability. As previously noted, clinical assessment typically relies on discrete variables, most commonly range of motion, or in more advanced evaluations, velocity. However, our findings show that isolated measures of range or peak velocity provide insufficient information on how activities of daily living are actually performed. Daily tasks inherently involve phases of deceleration and reacceleration, which allow for appropriate adjustment of cervical motion and contribute to overall coordination. The proposed model enables assessment of velocity throughout the entire movement trajectory, revealing attenuation during these dynamic phases in participants with higher disability. Consequently, intervention programs should not be restricted to increasing joint range of motion alone but should also incorporate dynamic training of acceleration (concentric contraction) and deceleration (eccentric contraction). This therapeutic approach targets the neuromuscular control of movement limits and enhances both efficiency and functional relevance of cervical motion.

This study had some limitations that should be acknowledged. The sample size was relatively modest, although sufficient to yield statistically significant results. Moreover, participants were restricted to patients with nonspecific neck pain and mild to moderate disability. The ability of the method to detect significant associations within such a narrow NDI range may be considered a strength, since correlations are usually stronger when the spectrum of disability is wider and includes severe cases, where kinematic differences are more pronounced [10,12,38]. Even so, larger and more heterogeneous cohorts will be necessary to confirm the stability of the findings and extend their generalizability. Within such cohorts, the potential influence of age and sex should also be examined in more detail, as the present sample was relatively young and unbalanced, which may have limited the detection of demographic effects on the results. In addition, the coefficient functions and their interpretation refer specifically to patients with mild to moderate nonspecific neck pain; other clinical conditions may present different kinematic–disability relationships that will need to be determined in future studies. Despite these limitations, the present work makes a methodological contribution by demonstrating how functional regression can be used to control overfitting, select the most relevant predictors, and identify the motion phases most closely related to disability.

## Conclusions

This study provides three methodological contributions to the assessment of neck disability using functional data analysis. First, scalar-on-function regression was applied to patients with nonspecific neck pain using kinematic curves instead of reduced summary measures. Second, a model selection strategy combining information criteria (AIC/BIC) with conventional fit indices was implemented to ensure accuracy and robustness. Third, a bivariate functional analysis was introduced, showing how functional regression can improve prediction and help identify which kinematic features are most closely associated with disability. Together, these contributions strengthen the methodological foundation and enhance the clinical interpretability of functional data analysis.

Our results, obtained in patients with nonspecific neck pain and low to moderate levels of disability, indicate that functional regression outperforms conventional models for assessing disability from cervical kinematics. This approach consistently identified angular velocities of flexion–extension and lateral flexion as the most informative predictors, and revealed that specific phases of motion—particularly braking and re-acceleration during flexion–extension—were associated with greater disability. Beyond predictive performance, these findings demonstrate the capacity of functional data analysis to uncover clinically meaningful patterns.

## Supporting information

S1 Data Kinematic data. The data is stored in an RData file which contains 4 lists: 1) FE: this list contains the matrices of the angle (FEfi) and angular velocity (FEdfi) of the neck flexion-extension movement. The matrices are 56x101, so each row corresponds to a subject (the total sample is 56). 2) FL: matrices of angle (FLfi) and angular velocity (FLdfi) of lateral flexion. The matrices are 56x101, so each row corresponds to a subject. 3) RA: matrices of angle (RAfi) and angular velocity (RAdfi) of axial rotation. The matrices are 56x101, so each row corresponds to a subject. 4) num: this list contains the scalar data for each subject, so they are vectors of length 56: NDI (NDI value), sex (coded as 0 = male, and 1 = female), age (in years) l_neck (neck length in cm), mass_head (head mass in kg) and mass_neck (neck mass in kg).
(ZIP)
